# *NDN* is an imprinted tumor suppressor gene that is downregulated in ovarian cancers through genetic and epigenetic mechanisms

**DOI:** 10.18632/oncotarget.6576

**Published:** 2015-12-12

**Authors:** Hailing Yang, Partha Das, Yinhua Yu, Weiqun Mao, Yan Wang, Keith Baggerly, Ying Wang, Rebecca T. Marquez, Anuja Bedi, Jinsong Liu, David Fishman, Zhen Lu, Robert C. Bast

**Affiliations:** ^1^ Departments of Experimental Therapeutics, U.T.M.D. Anderson Cancer Center, Houston, TX 77030, USA; ^2^ Departments of Bioinformatics, U.T.M.D. Anderson Cancer Center, Houston, TX 77030, USA; ^3^ Departments of Pathology, U.T.M.D. Anderson Cancer Center, Houston, TX 77030, USA; ^4^ Department of Gynecology, Obstetrics and the Gynecology Hospital, Fudhan University, Shanghai, PR China; ^5^ Texas College of Osteopathic Medicine, Fort Worth, TX 76107, USA; ^6^ Gynecology and Reproductive Science, Mount Sinai Hospital, New York, NY 10029, USA

**Keywords:** NDN, FAK, tumor suppressor gene, imprinting, growth inhibition, cell motility

## Abstract

*NDN* is a maternally imprinted gene consistently expressed in normal ovarian epithelium, is dramatically downregulated in the majority of ovarian cancers. Little or no *NDN* expression could be detected in 73% of 351 epithelial ovarian cancers. *NDN* was also downregulated in 10 ovarian cancer cell lines with total loss in 6 of 10. Re-expression of *NDN* decreased Bcl-2 levels and induced apoptosis, which significantly inhibited ovarian cancer cell growth in cell culture and in xenografts. In addition, re-expression of *NDN* inhibited cell migration by decreasing actin stress fiber and focal adhesion complex formation through deactivation of Src, FAK and RhoA. Loss of *NDN* expression in ovarian cancers could be attributed to LOH in 28% of 18 informative cases and to hypermethylation of CpG sites 1 and 2 of *NDN* promoter in 23% and 30% of 43 ovarian cancers, respectively. Promoter hypermethylation was also found in 5 of 10 ovarian cancer cell lines. Treatment with the demethylating agent 5-aza-2′-deoxycytidine restored *NDN* expression in 4 of 7 cell lines with enhanced promoter methylation levels. These observations support the conclusion that *NDN* is an imprinted tumor suppressor gene which affects cancer cell motility, invasion and growth and that its loss of function in ovarian cancer can be caused by both genetic and epigenetic mechanisms.

## INTRODUCTION

Oncogenic drivers of ovarian cancer have received considerable attention, but tumor suppressor genes have not been studied as fully. Among the tumor suppressor genes discovered to date in ovarian cancers, several are imprinted. As loss of autosomal suppressor function requires the loss of both alleles, imprinted genes are of particular interest, in that only a single allele is expressed and a single hit can silence function. *ARHI* (*DIRAS3*), a maternally imprinted gene, has been studied in depth and found to mediate cell growth [1], motility [2], autophagy and tumor dormancy [3]. *DIRAS3* can be downregulated by multiple mechanisms, including loss of heterozygosity and promoter hypermethylation [4]. We have found that other imprinted growth inhibitory genes are downregulated in ovarian cancer, including *NDN*. Consequently, we have examined the function of *NDN* and mechanisms of its downregulation to permit comparison with *DIRAS3* and other imprinted tumor suppressor genes.

*NDN* is a maternally imprinted gene. Genomic imprinting is an epigenetic mechanism to ensure that certain genes are expressed at carefully regulated levels in a parent-of-origin-specific manner, which is critical for normal growth and development [5]. Monoallelic expression of an imprinted gene is established and maintained by differential DNA methylation between the parental alleles [6]. *NDN* is located on chromosome 15q11.2 within a large cluster of imprinted genes including *MKRN3*, *MAGEL2*, *SNRPN*, and *UBE3A* [7]. Imprinting of this cluster is controlled by an imprinting center (IC) located 1.3Mb upstream of the *NDN* gene at the 5′ end of the *SNRPN* gene, including the *SNRPN* promoter and exon 1 [8].

The *NDN* gene encodes the necdin protein which is predominantly expressed in postmitotic neurons and is excluded from mitotic cells [9]. Studies of brain development suggest that necdin inhibits neuronal growth. Ectopic expression of necdin inhibits NIH3T3 cell growth and also suppresses clonogenic growth of SAOS-2 cells [10, 11]. *NDN* downregulation has been found in several diseases. *NDN* silencing by deletion, uniparental disomy or translocation is causally associated with the Prader-Willi syndrome, a congenital neuro-developmental disorder [12]. Down regulation of *NDN* has also been found in surgical specimens of prostate and urothelial cancers [13, 14] and in telomerase TERT immortalized human urothelial cells [15].

We have found that *NDN* is consistently expressed in normal ovarian surface epithelial cells but is frequently downregulated in surgical specimens of ovarian cancers and cancer cell lines. Whether *NDN* functions as a tumor suppressor gene in ovarian cancer or how it is downregulated has not previously been addressed. To evaluate its role in the function of ovarian cancers, we re-expressed *NDN* in SKOv3ip and HEY by transient transfection and also generated several inducible cell lines from SKOv3ip line. Re-expression of *NDN* inhibited cell growth and decreased cell motility and migration. Growth inhibition was associated with apoptosis, but not autophagy, senescence or necrosis. To investigate the possible genetic and/or epigenetic mechanism(s) of *NDN* silencing, we measured the DNA methylation status and LOH in normal ovarian tissue and in ovarian cancers. Both LOH and DNA hypermethylation contribute to *NDN* downregulation.

## RESULTS

### *NDN* expression in ovarian cancers and cancer cell lines is dramatically decreased

**Figure 1:**
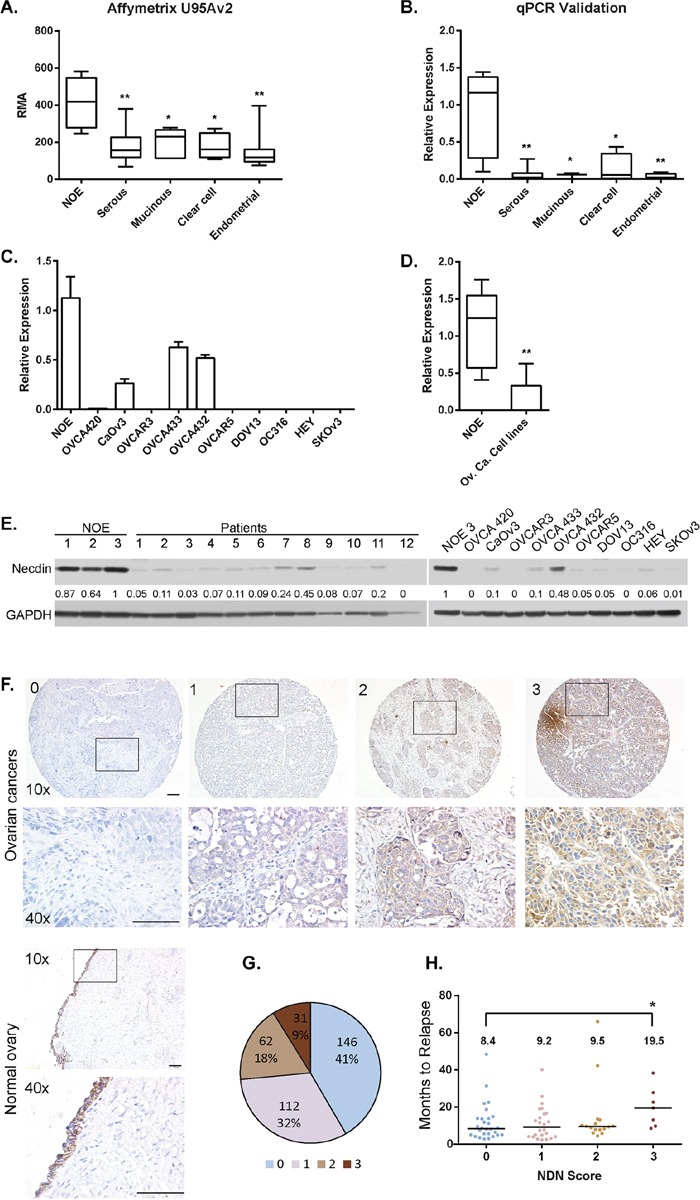
*NDN* expression is downregulated in ovarian cancers and cancer cell lines **A.** *NDN* mRNA expression in 35 ovarian cancers with different histotypes and 5 pools of normal ovarian epithelial scrapings (NOE) was profiled on Affymetrix U95Av2 array. **B.** Validation of microarray results using the same samples with qPCR. **C.** *NDN* expression levels were determined in NOE and 10 ovarian cancer cell lines using qPCR. **D.** Pooled *NDN* expression in 10 ovarian cancer cell lines. Data represent Mean ± S.D.. Asterisk denotes significant difference (* *p* < 0.05, ** *p* < 0.01) compared with NOE using a two-tailed student t test. **E.** Necdin levels in twelve high grade serous tumor samples and 10 cancer cell lines were measured by western blot analysis and compared to three normal ovarian epithelial samples. **F.** Normal ovaries and tumor tissue microarray with 351 interpretable cases were analyzed using immunohistochemistry with anti-necdin antibody and scored as 0 to 3. 4 examples from the tumor array representing the score 0 (no expression), 1 (low expression), 2 (moderate expression) and 3 (high expression) were shown with 10x magnification, and the insets were enlarged to 40x magnification. Bar is equal to 100 μm. **F.** The fraction of ovarian cancers with necdin expression. **G.** Correlation between disease free survival and necdin expression. Each circle represents one patient sample. The median disease free survival time was listed on top. Asterisk denotes significant difference (* *p* < 0.05) compared with 0 score.

To characterize gene expression patterns in ovarian cancers, Affymetrix microarray analysis was performed using 35 flash-frozen primary ovarian cancer specimens, comparing their gene expression to 5 pools of normal human ovarian surface epithelial scrapings (NOE). *NDN* is one of the genes downregulated more than 2 fold in all 4 histotypes (Figure 1A and Supplementary Table 3). The data was validated using quantitative PCR (Figure 1B). *NDN* expression levels in 10 ovarian cancer cell lines were also measured using qPCR, and compared with pooled NOE (Figure 1C and 1D). All 10 cell lines exhibited a dramatic reduction in *NDN* expression. Six of 10 cell lines lost *NDN* expression entirely, one cell line (OVCA 420) showed minimal detectable expression, and 3 cell lines (CAOv3, OVCA 432 and 433) exhibited 30-60% expression (Figure 1C). To determine protein expression in clinical specimens and human cancer cell lines, the levels of *NDN* gene product, necdin, were examined by western blot analysis (Figure 1E). We compared three primary cultures of normal ovarian epithelium with 12 cancer tissues. All cancers showed decreased necdin expression at different levels. Necdin levels were also reduced in all ten cancer cell lines, with similar patterns to mRNA levels. 10 examine necdin expression in a larger population of cancer patients, ovarian cancer tissue microarray was stained with a monoclonal necdin antibody, and compared with normal ovaries (Figure 1F and Supplementary Figure S2). Of 351 cases, 146 cancers (41%) did not express necdin, scored as 0; 112 cancers (32%) had definite but low expression, scored as 1; 62 cancers (18%) had moderate expression, scored as 2; and 31 cancers (9%) had high expression, scored as 3 (Figure 1G). Necdin levels were also analyzed by 4 major histotypes (Supplementary Figure S1B). For 234 serous cancers, 180 cases (76%) expressed minimal levels of necdin (score 0 and 1). For 21 endometrial cancers, 13 cases (62%) were negative or low expressers. For 5 mucinous cancers, none showed high necdin expression. For 9 clear cell cancers, 2 had intermediate and 4 had high expression. Thus, necdin expression was frequently and dramatically decreased in serous, endometrial and mucinous cancers. For clear cell cancers, high expression of necdin accounted for a larger fraction of cases. However, there were not enough cases to make a valid statistic comparison. For high grade serous cancer cases, patients with high necdin expression showed a longer disease-free survival compared with patients with relatively low necdin expression (median disease free survival = 19.5 months vs 8–9 months in Figure 1H).

### *NDN* expression correlates with *DIRAS3* and *PEG3* expression at the mRNA and protein levels

To compare *NDN* expression with other known imprinted tumor suppressor genes downregulated in ovarian cancers, microarray expression data using 35 samples from MD Anderson Gynecologic Tissue Bank (MDAGTB) and 295 samples from Australian Ovarian Cancer Study (AOCS) were analyzed using a Pearson Correlation. *NDN* mRNA levels from the two databases were significantly correlated with the levels of *DIRAS3* and *PEG3* with r = 0.7837 and 0.7224 from MDAGTB and r = 0.2068 and 0.3259 from AOCS (Supplementary Figure S3A-S3B). To compare protein levels, *DIRAS3* and *PEG3* were also stained and scored using successive slides from the same ovarian cancer tissue microarray. Necdin levels were significantly correlated with the levels of *DIRAS3* and *PEG3* using a Pearson Chi-Square test (Supplementary Figure S3).

### Necdin expression inhibits tumor cell growth *in vitro* and *in vivo*

**Figure 2:**
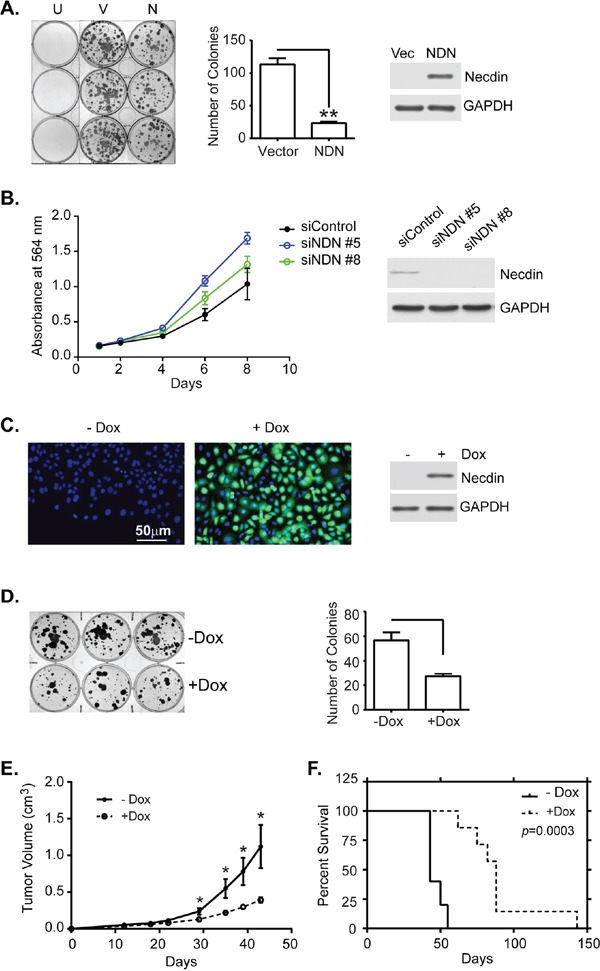
Necdin expression inhibits tumor cell growth *in vitro* and *in vivo* **A.** Transient expression of *NDN* inhibits colony formation. SKOv3ip cells were transfected with either empty vector (V) or a plasmid containing *NDN* cDNA (N). After 24 hrs, cells were reseeded into 6-well plates at a 1:16 dilution. G418 was added the next day to select transfected cells. Cells were incubated for 2 weeks. Untransfected cells (U) were eliminated by G418 selection. Colonies were stained with 0.5% methylene blue (Left panel), and counted (middle panel). Expression levels were measured using western blots (Right panel). **B.** Depletion of *NDN* increased cell growth in OVCA 432 cell line. OVCA 432 cells were transfected with *NDN* siRNA and incubated over time. Cells were fixed and stained with sulforhodamine B dye to examine cell viability. **C.** Necdin is expressed in SKOv3ip-*NDN* inducible cell line. The inducible cell line was treated with or without doxycycline (−/+Dox) for 48 hrs and then harvested for immunofluorescence (necdin in green and DNA in blue) and western blotting analyses. **D.** Necdin expression inhibits colony formation in an SKOv3ip-*NDN* inducible cell line. Six hundred cells were incubated in triplicate wells in a 6-well plate with or without DOX for 2 weeks. Colonies were stained (Left panel) and counted (Right panel). **E.** Necdin expression inhibits tumor growth in mouse xenograft model. Mice were injected subcutaneously with two million SKOv3ip-*NDN* inducible cells and were immediately fed with sucrose water with or without Dox for the duration of the experiment. Tumor volume was measured twice a week until tumor burden in the –Dox control group exceeded 2 cm requiring euthanasia. **F.** Necdin expression prolongs the survival of tumor bearing mice. The survival was determined at the time when tumor burden or ulceration reached to the limit. Asterisk denotes significance (* *p* < 0.05 and ** *p* < 0.01) by two-tailed student *t* test. Survival curves were generated using Kaplan-Meier estimate and compared by log-rank Mantel-Cox test (*p* < 0.001).

Ectopic expression of necdin in NIH3T3 and SAOS-2 cells suppressed cell growth [11]. To test whether re-expression of necdin affected growth of ovarian cancer cells, *NDN* cDNA was transfected into SKOv3ip and HEY cell lines that lacked endogenous necdin expression. Transient expression of necdin dramatically inhibited colony formation in both cell lines (Figure 2A for SKOv3ip and S4A for HEY). Depletion of *NDN* in OVCA 432, an ovarian cancer cell line with relatively high *NDN* expression improved cell proliferation (Figure 2B). To further study necdin's function in tumor suppression, Tet-on inducible cell lines from SKOv3ip line were generated by Applied Biological Materials (Richmond, BC Canada). Clone 3, 7 and 19 have approximately 80-90% cells expressing necdin upon doxycycline induction (Clone 7 shown in Figure 2C, and clone 3 and 19 in Supplementary Figure S2). Clone 7 was chosen for this study because the expression levels are more homogenous than those in the other two clones. The inducible cell line also showed a significant growth inhibition in clonogenic assays (Figure 2D). Tumor xenograft growth was inhibited and survival prolonged when necdin was induced by adding doxycycline to drinking water (Figure 2E and 2F).

### Necdin re-expression induces apoptosis by decreasing Bcl-2 mRNA

**Figure 3:**
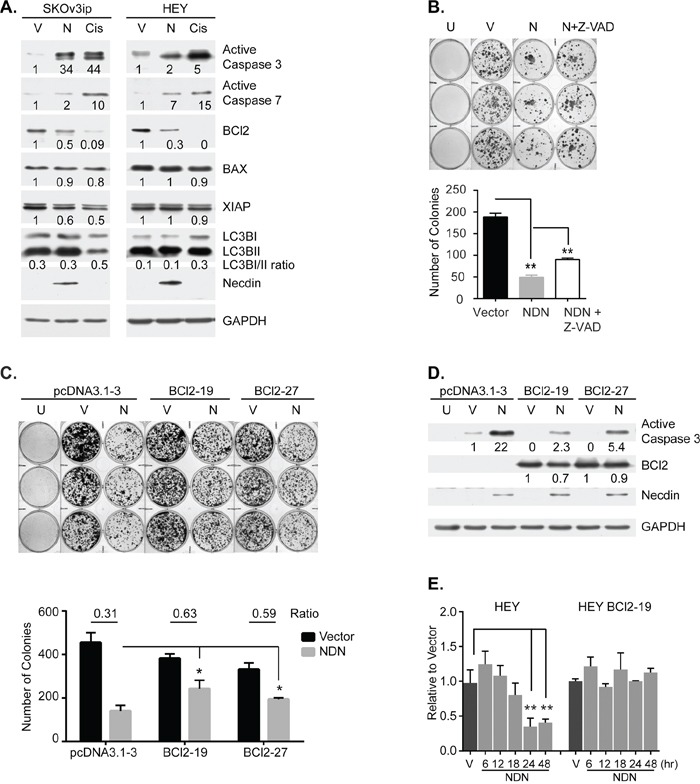
Necdin re-expression inhibits cell growth by inducing apoptosis **A.** Necdin expression induces apoptosis, but does not induce autophagy. SKOv3ip and HEY cells were transfected with either empty vector (V) or the plasmid containing *NDN* cDNA (N) for 48 hrs and then lysed for western blotting. Wild type cells treated with 50 μM cisplatin (Cis) for 48 hrs were included as positive control for apoptosis. **B.** Growth inhibition by *NDN* re-expression can be partially rescued by Z-VAD-FMK. SKOv3ip cells were pre-incubated with or without 20 μM pan caspase inhibitor, Z-VAD-FMK for 3 hrs, and then transfected with *NDN* or vector control. After 24 hrs, cells were reseeded into 6-well plates at 1:16 dilution. G418 was added the next day to select transfected cells. Cells were allowed to grow for 2 weeks. Media supplemented with G418 and Z-VAD-FMK were changed every other day. Untransfected cells (U) were killed by G418 selection. Colonies were stained (Top panel) and counted (Bottom panel). **C.** Growth inhibition by *NDN* re-expression can be partially rescued by Bcl-2 over-expression. Stable HEY cell lines expressing either control vector or Bcl-2 gene were transfected with empty vector or *NDN*. After 24 hrs, 1000 cells were reseeded into 6-well plates. 3 μM Blasticidin was added the next day to select transfected cells. Cells were allowed to grow for 1 week and then stained (Top panel). Colonies were scored and the ratio between vector and *NDN* was calculated (Bottom panel). **D.** Bcl-2 overexpression attenuates *NDN* induced apoptosis. Bcl-2 stable cell lines were transfected as described in C and harvested after 48 hrs. **E.** Necdin re-expression decreases Bcl-2 mRNA levels. WT HEY and HEY Bcl-2 stable clone 19 were transfected with empty vector or *NDN*. mRNA was extracted at 6, 12, 18, 24 and 48 hrs post transfection. Bcl-2 mRNA levels were determined by RT-qPCR. Asterisk denotes significance (* *p* < 0.05 and ** *p* < 0.01) by two-tailed student *t* test.

To determine how necdin inhibits cancer cell growth, we examined the effect of expressing necdin protein on cell survival. Apoptosis, autophagy, necrosis and senescence were measured in ovarian cancer cells transiently transfected with *NDN* cDNA or induced by exposure to doxycycline. Transient expression of *NDN* induced cleavage and activation of executioner caspases 3 and 7, indicative of apoptosis in ovarian cancer cells (Figure 3A). Necrosis, senescence or increased autophagy was not observed. Increased expression of *NDN* did not affect the ratio of LC3B-I to LC3B-II and did not increase the number of necrotic and senescent cells (Figure 3A, Supplementary Figures S5 and S6). A pan caspase inhibitor, Z-VAD-FMK, blocked caspase 3 and 7 activation and partially rescued necdin-induced growth inhibition (Figure 3B and Supplementary Figure S4B). Next, we examined the effect of necdin expression on three regulators of intrinsic caspase activation, anti-apoptotic Bcl-2, anti-apoptotic XIAP and pro-apoptotic BAX by western blotting. Necdin expression reduced Bcl-2 protein levels in both SKOv3ip and HEY cells but did not affect BAX or XIAP levels as drastically. To test whether changes in Bcl-2 expression might regulate necdin-induced apoptosis, we transfected *NDN* into sublines of HEY cells with stable expression of Bcl-2 [17]. Constitutive Bcl-2 expression attenuated the growth inhibition induced by necdin (Figure 3C) and decreased caspase 3 activation (Figure 3D). The results suggest that necdin expression induces apoptosis, at least in part, by decreasing Bcl-2 levels. Necdin expression significantly reduced Bcl-2 mRNA levels in wild type HEY cells soon after *NDN* transfection, but did not affect Bcl-2 mRNA in HEY cells with forced Bcl-2 expression from a CMV promoter (Figure 3E). Bcl-2 function and expression is regulated by phosphorylation. Phosphorylation of Bcl-2 S70 stabilizes interaction with BAX, and inhibits Bcl-2 degradation upon growth factor stimulation [18]. Phosphorylation of S87 protects Bcl-2 from ubiquitin-dependent degradation [19]. However, necdin expression did not alter the phosphorylation status of Bcl-2 in wild type HEY and HEY with forced expression of Bcl-2 (Supplementary Figure S7). When WT HEY cells were treated with a proteasome inhibitor MG-132, endogenous Bcl-2 levels were not affected. However, Bcl-2 levels in HEY cells with forced Bcl-2 expression were slightly increased. These observations are consistent with the possibility that necdin transcriptionally regulates Bcl-2 expression.

### Necdin re-expression inhibits cell motility by inhibiting Src, FAK and RhoA activity

**Figure 4:**
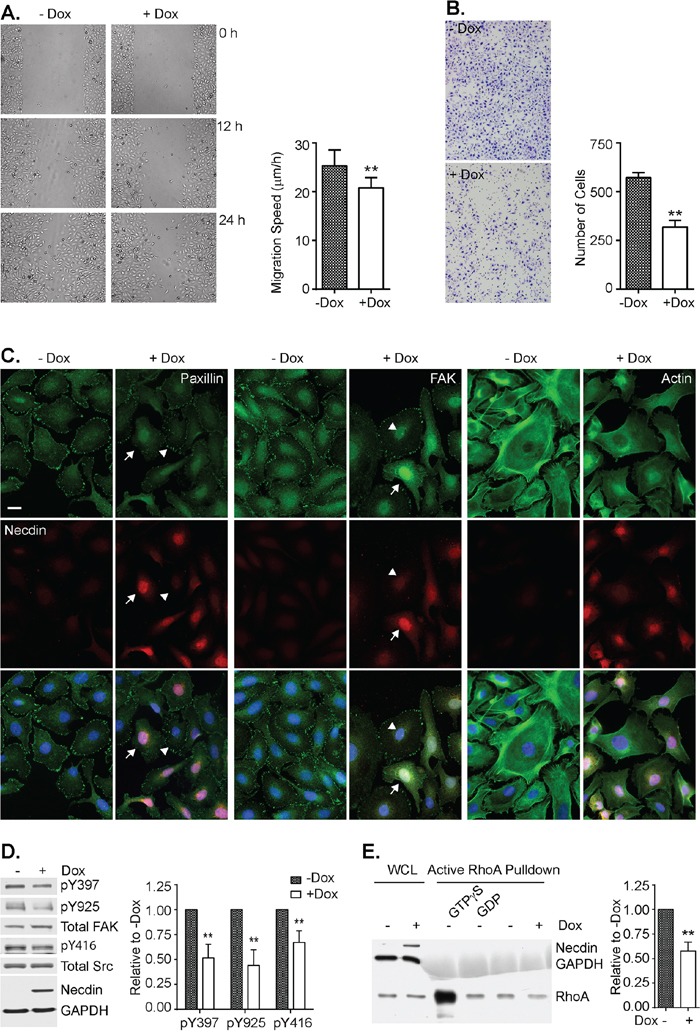
Necdin re-expression inhibits cell motility in SKOv3ip-NDN inducible cell line Cells were grown with or without Dox induction for 48 hrs and then treated for the following assays. **A.** *NDN* inhibits wound closure. Following a scratch, the wound closure was recorded every 6 hrs up to 36 hrs. The migration speed was calculated by dividing gap width over time. **B.** *NDN* inhibits invasion by Boyden chamber assay. After 48-hr Dox induction, 4,000 cells were resuspended in serum free media and transferred onto PET membrane with 8 μm pore. Serum media were placed on the other side of the membrane to create chemotaxis. Cells were stained after 16 hrs with HEMA3 staining kit. **C.** *NDN* reduces stress fiber and focal adhesion formation by immunofluorescence. After 48-hr Dox induction, cells were labeled with Oregon Green phalloidin and anti-nedcin antibody (Left panel), anti-paxillin and anti-necdin antibodies (middle panel), or anti-FAK and anti-necdin antibodies (Right panel), and viewed with epi-fluorescence microscope. Nucleus was counterstained with DAPI. High expressors were pointed by arrows and low expressors by arrow heads. **D.** *NDN* re-expression deactivates Src and FAK by Western Blot. Cells was induced by doxycycline for 48 hrs and then lysed and resolved in 8% SDS-PAGE gel. The blots were shown on the left. The experiment was repeated 4 times and analyzed with Image J software, shown on the right. **E.** *NDN* re-expression inhibits RhoA activity. Active RhoA was pulled down by GST-Rhotekin and run on 15% SDS-PAGE gel. Membrane was probed with anti-RhoA, anti-necdin, and anti-GAPDH antibodies. The Density of bands was measured by Image J and normalized to whole cell lysate (WCL). RhoA activity was averaged from three individual experiments and plotted in the right panel. Asterisk denotes significant difference (** *p* < 0.01) compared with –Dox group by two-tailed student *t* test.

As cell motility is essential for ovarian cancer metastasis, we used *NDN*-inducible cells to test the effect of necdin on cell motility and chemotaxis. Wound healing assays and Boyden chamber assays were performed to measure cell migration and invasion in the presence or absence of doxycycline. Necdin expression decreased the rate of cell migration, whereas necdin depletion by siRNA increased the migration rate (Figure 4A and S9). Necdin expression also decreased the average number of cells that crossed the membrane by 50% (Figure 4B). Focal adhesion and stress fiber formation, which are critical for cell motility, were examined by immunofluorescence microscopy (Figure 4C). Actin polymers were stained brightly with Oregon Green phalloidin and formed thick bundles when necdin was not expressed. Actin staining was decreased in cells that expressed necdin and actin bundles were less evident. Signals from focal adhesion proteins, such as focal adhesion kinase (FAK) and paxillin, were also drastically reduced when necdin was expressed. The levels of FAK and paxillin signals at focal adhesion were inversely related to necdin levels. FAK is activated through autophosphorylation at Tyrosine 397 (Y397). Activated FAK recruits Src, causing Src autophosphorylation at Tyrosine 416 (Y416) [20, 21]. Activated Src then phosphorylates FAK at Tyrosine 925 (Y925) [22]. Even though necdin re-expression did not alter FAK expression, it reduced active phosphorylated FAK levels by approximately 50% and inhibited Src activity by 30% (Figure 4D). Thus necdin downregulates FAK activity and prevents paxillin and FAK from assembling into focal adhesion complexes. As FAK function is mediated through the small GTPase RhoA [23], we measured its activity in the presence and absence of necdin using an active Rho pull-down and detection kit. Lysates pretreated with GTPgS or GDP served as positive and negative controls. Necdin expression inhibited RhoA activity by 50% (Figure 4E). These data suggested that necdin expression inhibited ovarian cancer cell motility via Src – FAK – RhoA pathway.

### Loss of heterozygosity (LOH) of *NDN* gene occurs in 28% of ovarian cancers

**Figure 5:**
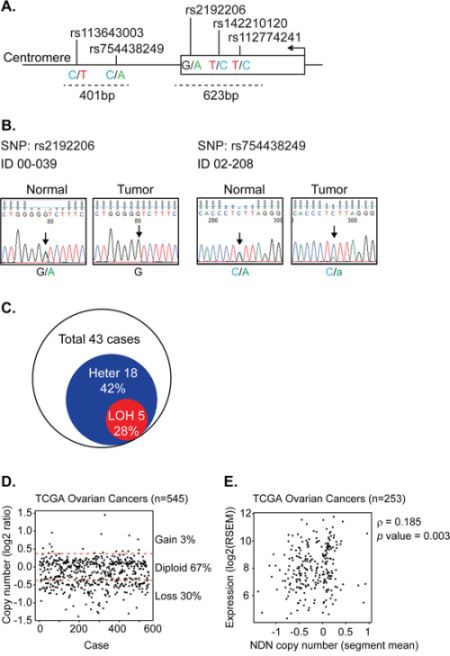
Loss of heterozygosity of *NDN* is found in a fraction of ovarian cancers **A.** Strategy for LOH detection using SNP genotyping. The arrow indicates the transcription start site and direction. The dotted lines indicate the size and location of the PCR products containing 5 SNPs. **B.** Two examples of LOH detected in paired patient samples using Sanger based sequencing for rs2192206 and rs754438249. The arrows point out the SNPs with double peaks in the normal tissue which turned to a single peak or a smaller peak in the tumor tissue from the same patient, suggesting loss of one allele. **C.** Fraction of ovarian cancers with LOH in 43 cases. **D.** Copy number variation from TCGA database. **E.** The correlation between copy number and gene expression using the Spearman's rank correlation test.

To identify mechanisms by which *NDN* is downregulated, we genotyped 5 single nucleotide polymorphisms (SNPs) along *NDN*'s coding region and 3′-UTR (Figure 5A) in the same 43 paired normal and cancer samples (Supplementary Table S4). Among the 5 SNPs, we only detected mutations in SNP rs2192206 and rs754438249. The frequencies in normal tissues were 35% (15 of 43 cases) and 12% (5 of 43 cases), respectively. Thus, 18 normal tissues (42%) were informative heterozygotes. Five matched cancer tissues (28%) lost heterozygosity (Figure 5B–5C). For the other three SNPs, no mutations were detected. We also searched the ovarian cancer database by the Cancer Genome Atlas program (TCGA) and found that 30% of 545 tumors showed copy number loss (Figure 5D). The copy number change also showed a weak correlation with *NDN* expression (Figure 5E). These data indicate that LOH and copy number loss also contribute to *NDN* down-regulation.

### Promoter hypermethylation occurs in a majority of ovarian cancer cell lines and a fraction of primary ovarian cancers

**Figure 6:**
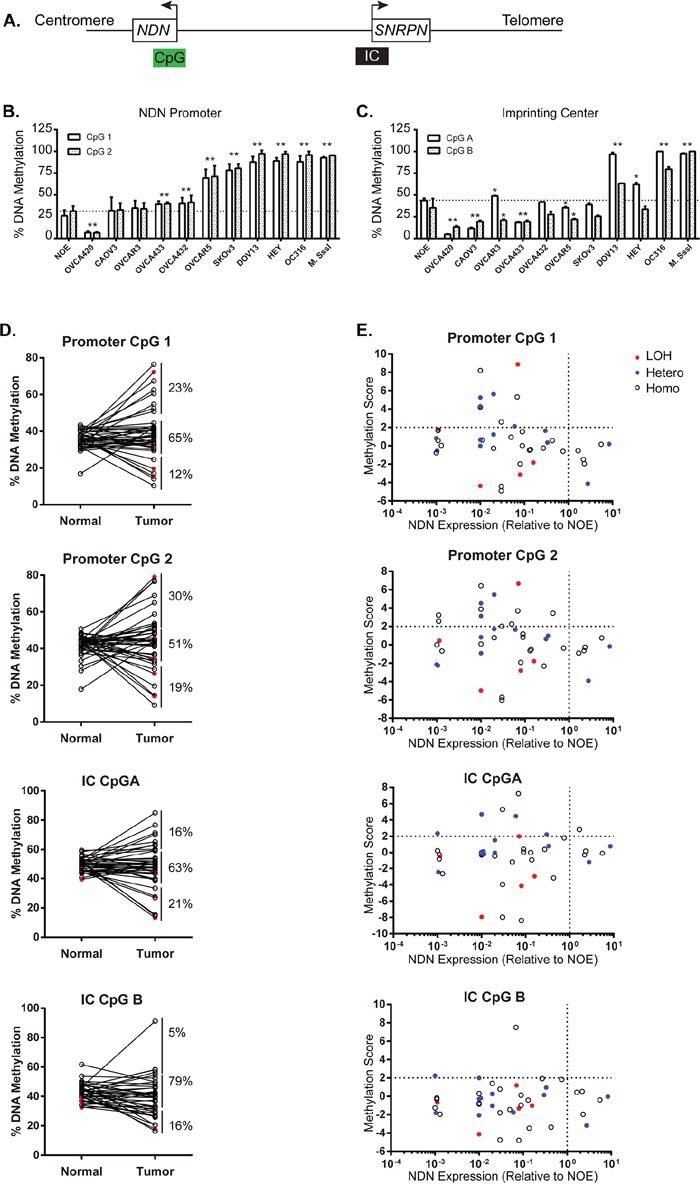
Aberrant NDN Methylation is found in a fraction of ovarian cancers and cancer cell lines **A.** A schematic diagram of the CpG islands in the *NDN* promoter and coding region, and the upstream imprinting center (IC). **B.** Methylation of 2 CpG sites 1 and 2 in the *NDN* promoter region in NOE and 10 ovarian cancer cell lines by pyrosequencing. **C.** Methylation of 2 CpG sites A and B in the imprinting center region. DNA pretreated with CpG methyltransferase (M. SssI) and S-adenosylmethionine served as the positive control. **D.** DNA methylation in 43 paired normal tissues and ovarian cancer samples. **E.** Correlation of DNA methylation, loss of heterozygosity, and expression. Methylation score was calculated for each pair using the following formula: Tumor-Normal/standard deviation of normal. Samples 2 S.D. above normal were considered hypermethylated. Open circles represent homozygous cases, solid blue circles represent informative heterozygous cases with no deletion, and solid red circles represent informative cases with LOH.

To determine whether DNA methylation contributes to *NDN* downregulation, DNA methylation was evaluated in 5 normal ovarian epithelial scrapings, 10 ovarian cancer cell lines, and 43 pairs of matched normal and primary ovarian cancer specimens. As an imprinted gene, *NDN*'s monoallelic expression is controlled by an imprinting center (IC) 1.3Mb upstream in the promoter and coding region of *SNRPN* gene. In the promoter and coding region of *NDN* gene, there is also a CpG island which can potentially be methylated and then regulate gene expression. Consequently, we designed 2 pairs of primers targeting both CpG islands in the *NDN* promoter and IC region, and probed 2 adjacent CpG sites for each region (Figure 6A). For the promoter region (Figure 6B), when compared with NOE, five cell lines, OVCAR5, DOV13, OC316, HEY and SKOv3ip, were intensively hypermethylated. Two cell lines, OVCA 433 and OVCA 432, had slightly increased methylation. CaOv3 and OVCAR3 showed normal levels of methylation compared with NOE. OVCA 420 was the only cell line which was hypomethylated. Two CpG sites (1 and 2) in the promoter region were altered concordantly. The methylation status in IC region was rather diverse (Figure 6C). The methylation levels in NOE were close to 50%, consistent with genomic imprinting. In 2 cell lines, DOV13 and OC316, both CpG A and B were hypermethylated. In OVCAR3 and HEY, CpG A was hypermethylated while CpG B was hypomethylated. In OVCA 420, CaOv3 and OVCA 433, the two CpGs were both hypomethylated. In OVCA 432, OVCAR5 and SKOv3ip, only CpG B was altered with decreased methylation.

For the 43 paired normal and tumor tissue samples, the average percentages of methylation for CpG 1 and 2 in the *NDN* promoter in normal tissues were 36% and 42%, respectively (Supplementary Table S5 and S6). The average percentages of methylation in paired cancer tissues were slightly increased to 39% and 44%, but the difference was not statistically significantly. For CpG 1, 23% (10 out of 43 cases) of cancer tissues were hypermethylated at least 2 standard deviation above paired normals; for CpG 2, 30% (13 out of 43 cases) were hypermethylated (Figure 6D). The average percentages of methylation for CpG A and B in the imprinting center in normal tissues were 50% and 43%, respectively, as expected for genomic imprinting. But the averages for cancer tissues were slightly, but not significantly, decreased to 48% and 39%, with only 16% hypermethylation cases in CpG A and 5% in CpG B.

### Promoter hypermethylation correlates with decreased gene expression

*NDN* was silenced in 5 ovarian cancer cell lines with heavy methylation in their *NDN* promoter regions. Among the 5 cell lines, two also had increased methylation in imprinting center. In ovarian cancer tissues from 43 patients, all cases with promoter hypermethylation showed decreased *NDN* mRNA levels (Figure 6E, top left quadrant). As for the imprinting center, 6 of 7 cases with CpG A hypermethylation had *NDN* downregulated. CpG B hypermethylation only occurred in 2 cases.

### *NDN* mRNA can be up-regulated by a demethylating agent

**Figure 7:**
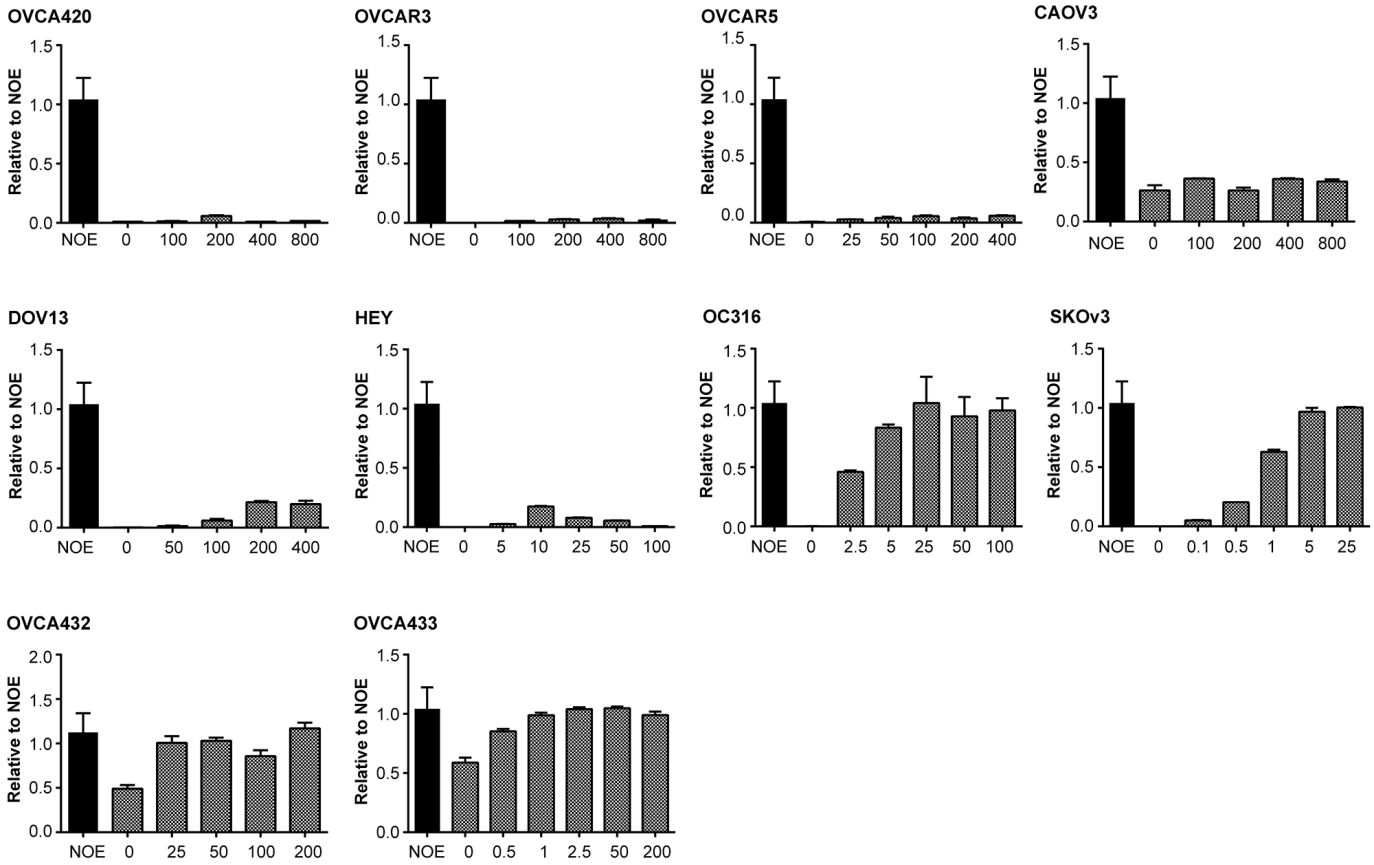
Treatment with a DNA demethylating agent (DAC) increases NDN gene expression at different levels in ovarian cancer cell lines 10 cell lines were treated with DAC and/or SAHA for 5 days. Fresh media with drugs was changed every day. *NDN* expression was measured by qPCR. Experiments were repeated three times.

To determine whether aberrant epigenetic modifications regulate *NDN* expression in ovarian cancers, the same 10 ovarian cancer cell lines were treated with a demethylating agent 5-aza-2′-deoxycytidine (DAC) (Figure 7). For seven cell lines with enhanced promoter methylation, DAC fully restored *NDN* expression in OC316, SKOv3, OVCA432 and OVCA433, and partially restored *NDN* expression in DOV13 and HEY, whereas it had minimal effect on OVCAR5. For cell lines with normal and low methylation status (OVCAR3, CAOV3 and OVCA420), DAC had no effect on *NDN* expression, suggesting that promoter hypermethylation plays important role for silencing *NDN* gene.

## DISCUSSION

Our data documents for the first time that re-expression of *NDN* inhibits growth and motility of ovarian cancer cells. Like *PEG3* [4, 24], but unlike *DIRAS3* [3], re-expression of *NDN* induces apoptosis. Again, in contrast to *DIRAS3*, necdin re-expression fails to induce autophagy and necrosis. Both *NDN* and *DIRAS3* inhibit motility and chemotaxis, by inhibiting Src, FAK and RhoA [2].

Necdin facilitates apoptosis by downregulating anti-apoptotic Bcl-2 and activating caspases. Re-expression of necdin decreases Bcl-2 mRNA expression, but has no effect on Bcl-2 phosphorylation at residues S70 and S87, which are critical for Bcl-2 function and proteasomal degradation. A proteasome inhibitor MG-132 failed to restore Bcl-2 protein levels in HEY cell lines. Previous studies have shown that necdin binds to the transactivation domain of E2F1 at the carboxyl terminus to repress E2F1 transactivation. [11]. E2F1 is a well-established Bcl-2 transactivator. Overexpressing E2F1 induces Bcl-2 expression while depleting E2F1 does the reverse [25, 26]. Necdin is located in the nucleus and is proposed as a transcription factor to repress E2F1. These observations indicate that necdin transcriptionally downregulates Bcl-2 expression and subsequently facilitates caspase activation.

Cell motility is a coordinated procedure which requires extracellular stimuli being transmitted to cytoskeletal components, mostly actin fibers through multiple routes. Focal adhesion kinase (FAK) is the hub that interacts with several signaling pathways. Upon activation, FAK undergoes autophosphorylation at Y397, recruits Src, acquires phosphorylation at Y925 and assembles focal adhesion complexes at contact sites. The complexes can then activate other regulatory molecules such as RhoA, a small GTPase in the Rho family. RhoA reorganizes actin stress fiber and regulates focal adhesion formation [27–30]. Necdin expression reduces FAK phosphorylation levels, indicating a reduction in FAK activity (Figure 4D). RhoA is also deactivated after necdin expression (Figure 4E). Morphologically, we observed that stress fiber formation and focal adhesion assembly were greatly impaired by necdin expression (Figure 4C). As a result, the cancer cell's ability to migrate and invade is largely abrogated by necdin expression.

Here we also report for the first time that *NDN* is dramatically downregulated in each of the ovarian cancer cell lines tested and in 73% of surgical specimens through both genetic and epigenetic mechanisms. Consistent loss in cell lines is of interest. Traditionally, ovarian cancer cell lines can be established from only a small fraction of clinical specimens. Loss of imprinted tumor suppressor function for *NDN*, *DIRAS3* and *PEG3* might permit growth of cancer cells in culture.

Downreuglation of *NDN* in nearly three-fourths of clinical specimens is also likely to be important. As a maternally imprinted gene, only the single paternal allele is expressed in normal tissues. We have found that loss of *NDN* expression related both to LOH and to promoter hypermethylation. LOH was found in 28% of informative ovarian cancer cases. Promoter hypermethylation is recognized as a common pathway for silencing tumor suppressor genes. NDN downregulation was associated with hypermethylation of promoter CpG sites 1 and 2 in 23% and 30% of cases, respectively (Figure 6D and Supplementary Table S5 and S6). *NDN* expression was downregulated in all cases with hypermethylation (Figure 6E). Promoter hypermethylation is more dramatic in ovarian cancer cell lines with 50% of the cell lines hypermethylated (Figure 6B). Imprinting center methylation is critical for programing genomic imprinting event, but it may not be as important for *NDN* downregulation since only a tiny fraction of ovarian cancers exhibited IC hypermethylation (Figure 6D). Correlation of the expression of *NDN*, *DIRAS3* and *PEG3* is remarkable. This could relate to promoter methylation in all three genes during aging and carcinogenesis.

*NDN* expression is regulated by multiple mechanisms including LOH, DNA methylation in the promoter region. For autosomal genes, loss of tumor suppressor function requires loss function for both alleles. As imprinted genes have only one functional allele, hypermethylation and LOH that lead to homozygous methylation can provide the additional hit to silence an imprinted tumor suppressor gene. However, not all the cases with *NDN* depletion can be explained by the mechanisms tested in this paper. It is possible that its expression is also regulated at transcriptional and translational level by transcription factors and microRNAs which need to be studied in the future.

## MATERIALS AND METHODS

### Gene expression array analysis

As previously reported, gene expression analysis was carried out on 35 flash frozen primary epithelial ovarian cancers and 5 pools of normal ovarian surface epithelial scrapings [16]. RNA was extracted using the RNeasy kit (Qiagen, Valencia, CA) and Affymetrix Human U95Av2 arrays were used to measure gene expression. The probe ID for *NDN* is 36073_at based on GenBank sequence U35139.

### Ovarian cancer cell lines and tissue samples

Ten human ovarian cancer cell lines (CaOv3, DOV13, HEY, OC316, OVCA 420, OVCA 432, OVCA 433, OVCAR3, OVCAR5, SKOv3ip) and 5 pools of normal human ovarian surface epithelial scrapings were grown at 37°C in an atmosphere of 5% CO_2_ and 95% air using the recommended cell culture media (Supplementary materials). Tet-on inducible cell lines from SKOv3ip line were generated by Applied Biological Materials (Richmond, BC, Canada). Sublines of HEY cells with stable expression of Bcl-2 were established as previously reported [17]. Forty three pairs of human epithelial ovarian cancer tissue and normal uterus from the same patients were obtained from the MD Anderson Cancer Center Gynecological Tissue Bank. Five pools of normal ovarian surface scrapings were obtained from the Division of Gynecologic Oncology at New York University using IRB approved protocols. All cancer and normal uterine specimens were snap frozen in liquid nitrogen and stored at −80°C. Normal ovarian scrapings were stored at −20°C in RNAlater® (Ambion, Foster City, CA). Ovarian cancer tissue microarrays were prepared by the Pathology Core of the M.D. Anderson SPORE in Ovarian Cancer. The microarrays contained 412 distinct ovarian cancer specimens of which 351 were epithelial ovarian cancers where immunohistochemical staining could be interpreted. All the protocols and informed consents were approved by the respective Institutional Review Boards.

### RNA extraction, cDNA synthesis and quantitative PCR

RNA from tumor tissues, pooled ovarian scrapings, and ovarian cancer cell lines was extracted using the RNeasy kit (Qiagen, Valencia, CA). cDNA was synthesized from 1 μg of RNA using the Superscript II® First Strand Synthesis Kit (Invitrogen, Carlsbad, CA). SYBR green based quantitative PCR was used to measure RNA levels. Relative expression was calculated by the 2^−ΔΔCT^ method using glyceraldehydes-3-phosphatase dehydrogenase (*GAPDH*) as the reference gene. The experiments were done at least twice and samples were measured in triplicate. Primers included: *NDN* forward 5′-GTAAGCTTGGTTGGTGCTTTCGCT and reverse 5′-TCCAAACTCTGCAGGAGCAGTCTA; *GAPDH* forward 5′-TCGACAGTCAGCCGCATC TTCTTT and reverse 3′-ACCAAATCCGTTGACTCCGACCTT.

### Immunohistochemical analysis

Formalin-fixed, paraffin-embedded ovarian cancer tissue microarrays were deparaffinized and rehydrated. The slides were steamed in 1x Diva decloaker for 10 min to retrieve latent epitopes, followed by sequential blocking steps with PeroxAbolish, Avidin and Biotin, and 5% bovine serum albumin (BSA) in phosphate buffered saline (PBS). The slides were incubated with a mouse anti-necdin antibody overnight at 4°C. The slides were washed and stained with a biotin-labeled 4plus anti-mouse secondary antibody for 30 min at room temperature followed by 4plus streptavidin horseradish peroxidase for 10 min. The slides were developed using a DAB chromogen kit and then counterstained with hematoxylin. Normal ovarian surface epithelial cells and *NDN* transfected cells were used as positive controls, and mouse IgG2a kappa and GST-necdin blocking as negative controls (Supplementary Figure S1). Staining reagents were from Biocare Medical and necdin antibody, isotype control and purified GST-necdin were purchased from Novus Biologicals (Littleton, CO). For tumor tissue microarrays, the slides were read separately by two investigators. Cases in which <20% of the core contained tumor were excluded from the analysis. Images were captured using an Olympus IX71 microscope equipped with a DP72 camera (Olympus, Center Valley, PA).

### Clonogenic assays

Human necdin cDNA in a pCMV6-AC vector (OriGene, Rockville, MD) was transfected into SKOv3ip and HEY ovarian cancer cells using MegaTran 1.0 transfection reagent (OriGene). Twenty four hours post transfection, cells were reseeded in 6-well plates and selected with G418 media (1.2 mg/ml for SKOv3ip and 0.7 mg/ml for HEY). The cells were allowed to grow for 12 days until visible colonies were formed and then stained with 0.5% methylene blue. Colonies containing at least 50 cells were scored.

### Small interfering RNA transfection

siRNA and transfection reagent were purchased from GE Dharmacon (Lafayette, CO). Two human *NDN* siRNA oligos #5 (target sequence 5′-CGCACGAGCUCAUGUGGUA-3′) and oligo #8 (target sequence 5′-GGGCUGCACCUGAGGCUAA-3′) were used to deplete human *NDN*. ON-TARGETplus non-targeting siRNA #2 (sequence 5′-UGGUUUACAUGUUGUGUGA-3′) was used as control. siRNA was incubated with DharmaFECT 4 for 20 min at room temperature and added to 96-well plates for cell viability assay or 6-well plates for wound healing and Western Blot assays. The final concentration for siRNA was 25 nM and 1.5% for DhamarFECT 4.

### Sulforhodamine B cell viability assay

OvCa432 cell line was reversely transfected with control and *NDN* siRNA in 96-well plates. Cells were fixed with 10% (V/V) trichloroacetic acid at 4°C for 20 min and stained with 0.1% (W/V) sulforhodamine B in 1% (V/V) acetic acid for 30 min. The excessive dye was washed with 1% acetic acid. The protein bound dye was dissolved in 10 mM Tris pH 8.0 for absorbance determination at 564 nm using Synergy II plate reader (BioTek, Winooski, VT).

### Wound healing assay

Inducible cells were grown to confluent with or without 1 μg/ml doxycycline for 48 hrs. OvCa432 cells were incubated with siRNA liposome for 48 hrs. Cell monolayer was scratched using a toothpick. Then the wound closure was recorded at 6-hr interval using a phase contrast Olympus IX71 microscope and XM10 camera (Olympus, Center Valley, PA). Vertical distance between two edges of wound was measured using cellSens Dimension imaging software by Olympus.

### Boyden chamber assay

Inducible cells were pretreated with or without doxycycline for 48 hrs. Cells were resuspended in serum-free medium and transferred into the upper compartment of Boyden chambers with membranes of 8.0 μm porosity. The lower compartment was filled with normal culture medium to serve as a chemoattractant. After 16-hr incubation, cells were fixed and stained with Diff-Quick reagents (Siemens, Deerfield, IL). Cells on the upper side of the membrane were wiped off before counting. Experiments were set in triplicate and repeated three times.

### Immunofluorescence microscopy

Cells grown on coverslips were fixed by 4% formaldehyde in PBS, penetrated with 0.1% Triton X 100 in PBS, and blocked with 3% BSA in PBS. Cells were incubated with primary antibody at 37°C for 2 hrs, washed with PBS, and probed with either Alex 488 or Alex 594 conjugated secondary antibody at 37°C for 1 hr. Detailed information for antibodies is listed in Supplemental materials. Cells were mounted on Gel Mount and viewed under an Olympus IX71 microscope with an XM10 camera.

### Active RhoA pull-down assay

The *NDN* inducible cell line was incubated with or without doxycycline for 48 hrs and lysed following the manufacturer's instruction (Catalog 16116 by Pierce, Rockford, IL). 500 μg lysate along with 500 μg GTPgs or GDP pretreated lysates were shaken with glutathione agarose resin in the presence of GST-Rhotekin Rho binding domain for 1 hr at 4°C. Active RhoA bound to resin was washed and then eluted for western blotting.

### Mouse xenograft studies

Two million *NDN*-inducible cells and the parental SKOv3ip line were injected subcutaneously into 6–week old female athymic nude mice from Harlan Laboratories. Each group contained 6 mice. 2 mg/ml Doxycycline in 5% sucrose or sucrose alone water was fed on the day of injection. Tumor width and length were measured twice a week using a caliper. Two independent measurements were averaged. The tumor volume was calculated by (width)^2^ × length/2. All procedures were carried out according to the animal protocol approved by the Institutional Animal Care and Use Committee of the MD Anderson Cancer Center at the University of Texas.

### SNP genotyping and LOH analysis

Five SNPs were studied using PCR amplification and Sanger based sequencing. Three SNPs in the coding region (rs112774241, rs142210120 and rs2192206) were amplified using forward primer 5′-GTGAGGGTCAGAAACCATTCA and reverse 5′-TTCTTGTAGCTGCCGATGAC; two SNPs in 3′-UTR (rs754438249 and rs113643003) were amplified using forward 5′-GGCCTGTTGCAAGGATTAAAG and reverse 5′-CATTCCCAACCATACTGCTTTG. Amplicons were sequenced with both forward and reverse primers. LOH frequency is expressed as a percentage of samples with LOH divided by the total number of informative cases.

### DNA extraction, bisulfide treatment and pyrosequencing

Genomic DNA was extracted using the QIAamp DNA Mini Kit (Qiagen, Valencia, CA). 0.5–2 μg of DNA was used for bisulfite modification. 2 μl of bisulfite modified DNA was used to prepare biotinylated PCR products which were then sequenced using Pyro Gold reagents and PSQ ™HS 96A Pyrosequencing System (Qiagen, Valencia, CA) according to manufacture instruction. Primers included: Promoter forward 5′-Gttgggggtttaggttgtaaagttag, reverse 5′-taaacccaaaaaccctacccttacc, and sequencing 5′ – atttttattttgttttgatatg, ImC forward 5′ – GTAGATATG TTAGAATAAAGTAAGGATTTG, reverse 5′ – AACCC CAAAACTACTATACACCTC, and sequencing 5′ – TTTGA GAGATTTTAATTTTGT, and biotinylated universal primer 5′-biotin-GGGACACCGCTGATCGTTTA.

### Treatment with a demethylating agent

Ovarian cancer cell lines in 60-mm plates were treated with different concentrations of 5-aza-2′-deoxycytidine (Sigma) for 5 days. Media were changed every day with fresh drug added. Cells were washed twice with PBS and RNA was extracted using TRIzol® reagent (Life Technologies, Grand Island, NY).

### Statistical analysis

Data are expressed as mean ± SD, unless otherwise specified. Statistical significance was analyzed using a two-tailed student's *t* test. Survival analysis was performed using the Kaplan-Meier method. A *p* < 0.05 was considered significant.

## SUPPLEMENTARY FIGURES AND TABLES




