# Supplementary material for: NDN is an imprinted tumor suppressor gene that is downregulated in ovarian cancers through genetic and epigenetic mechanisms
Source: Oncotarget. 2015 Dec 12;7(3):3018–32. doi: 10.18632/oncotarget.6576 (PMC4823087; doi:10.18632/oncotarget.6576)
Supplement: Supplementary file 1 [file oncotarget-07-3018-s001.pdf]

SUPPLEMENTARY FIGURES AND TABLES

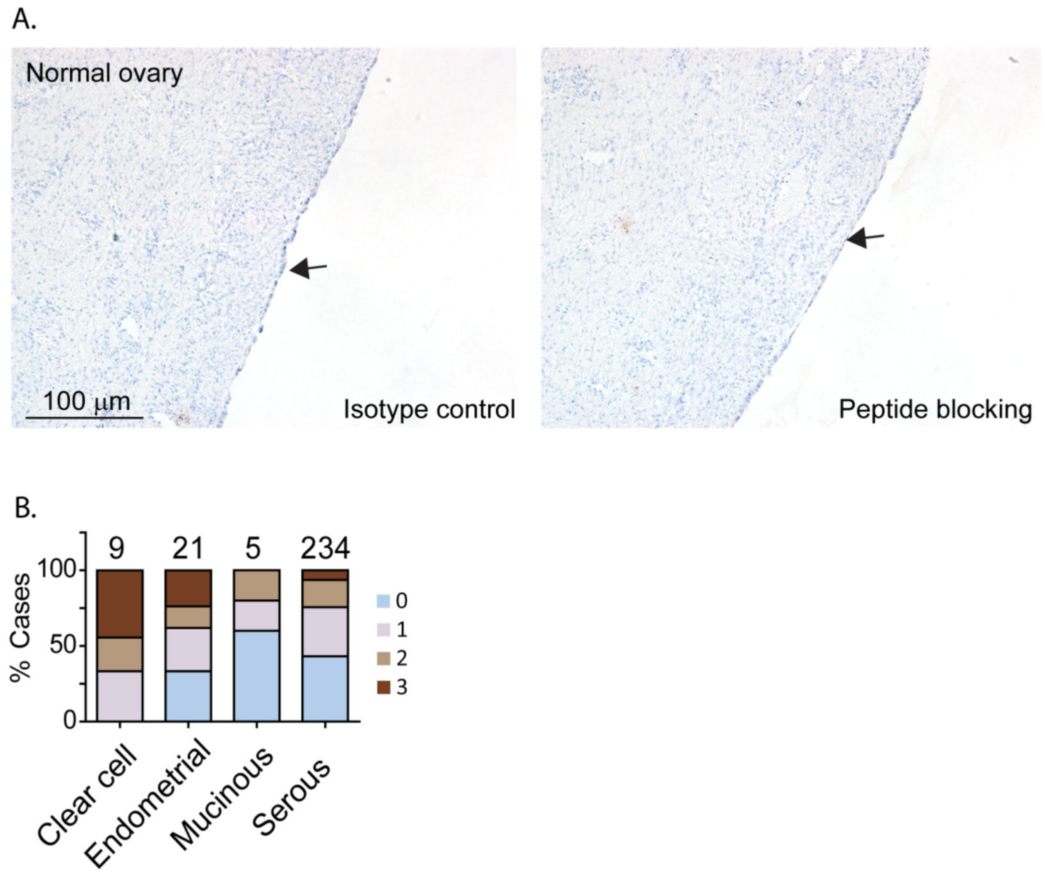

Supplementary Figure S1: A. Negative controls for anti-Necdin staining. Left, IgG2a kappa isotype control. Right, Necdin blocking. Anti –Necdin antibody was pre-incubated with purified GST-fused Necdin protein for 3 h before probing. Arrows point to ovarian surface epithelial layer. **B.** The distribution of necdin expression in 4 major ovarian cancer histotypes.

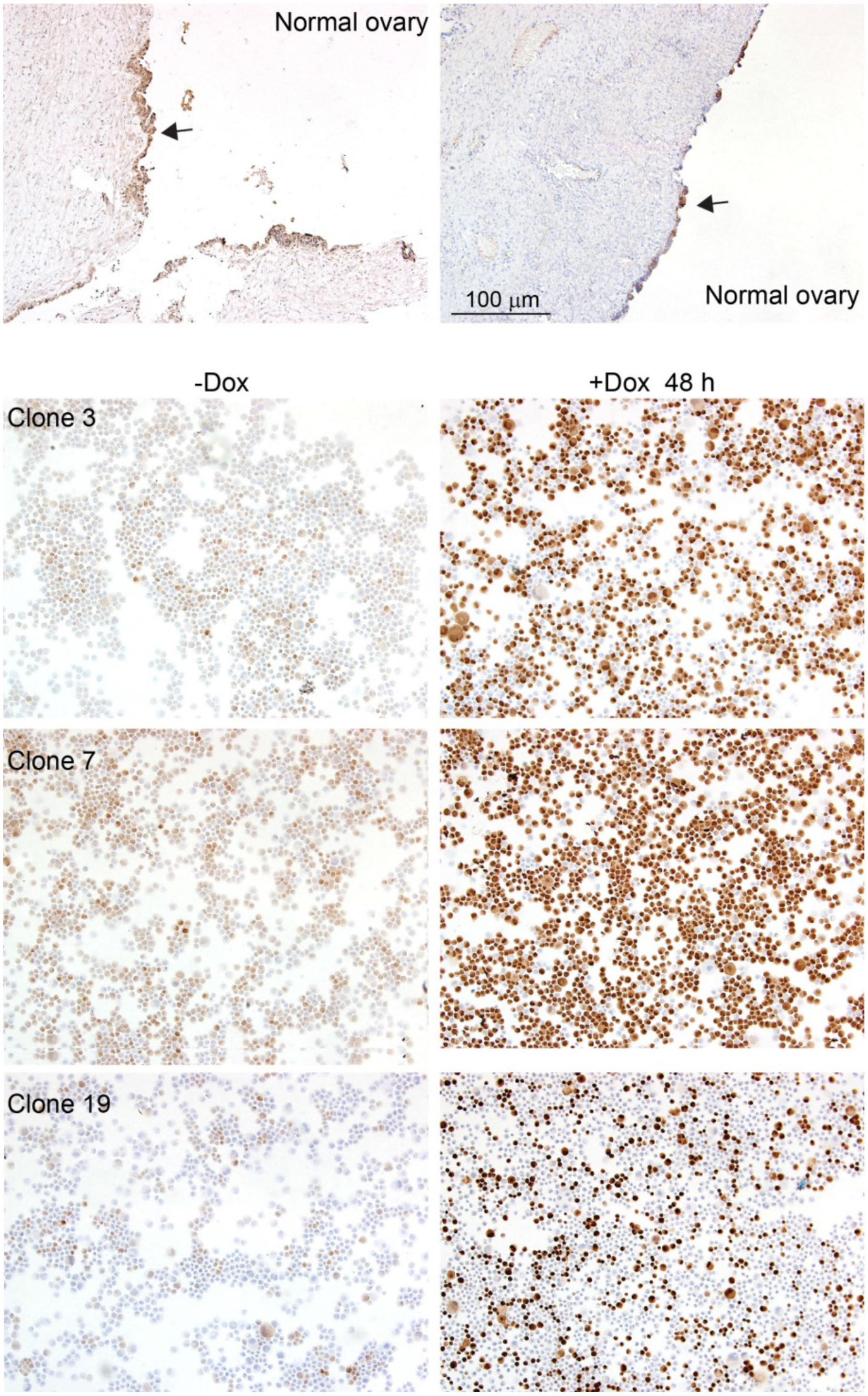

Supplementary Figure S2: Necdin expression in normal ovaries and 3 inducible cell lines. Normal ovary tissues were formalin fixed paraffin embedded. SKOv3ip *NDN* inducible cell lines clone 3, 7 and 19 were incubated with or without Dox for 48 h. Cells were trypsinized, spun onto (+) slides at 800 g for 5 min and air dried overnight. Cells were fixed with 4% formaldehyde in PBS for 30 min followed by 1-hr ethanol penetration at −20°C. Standard immunohistochemistry protocol was utilized.

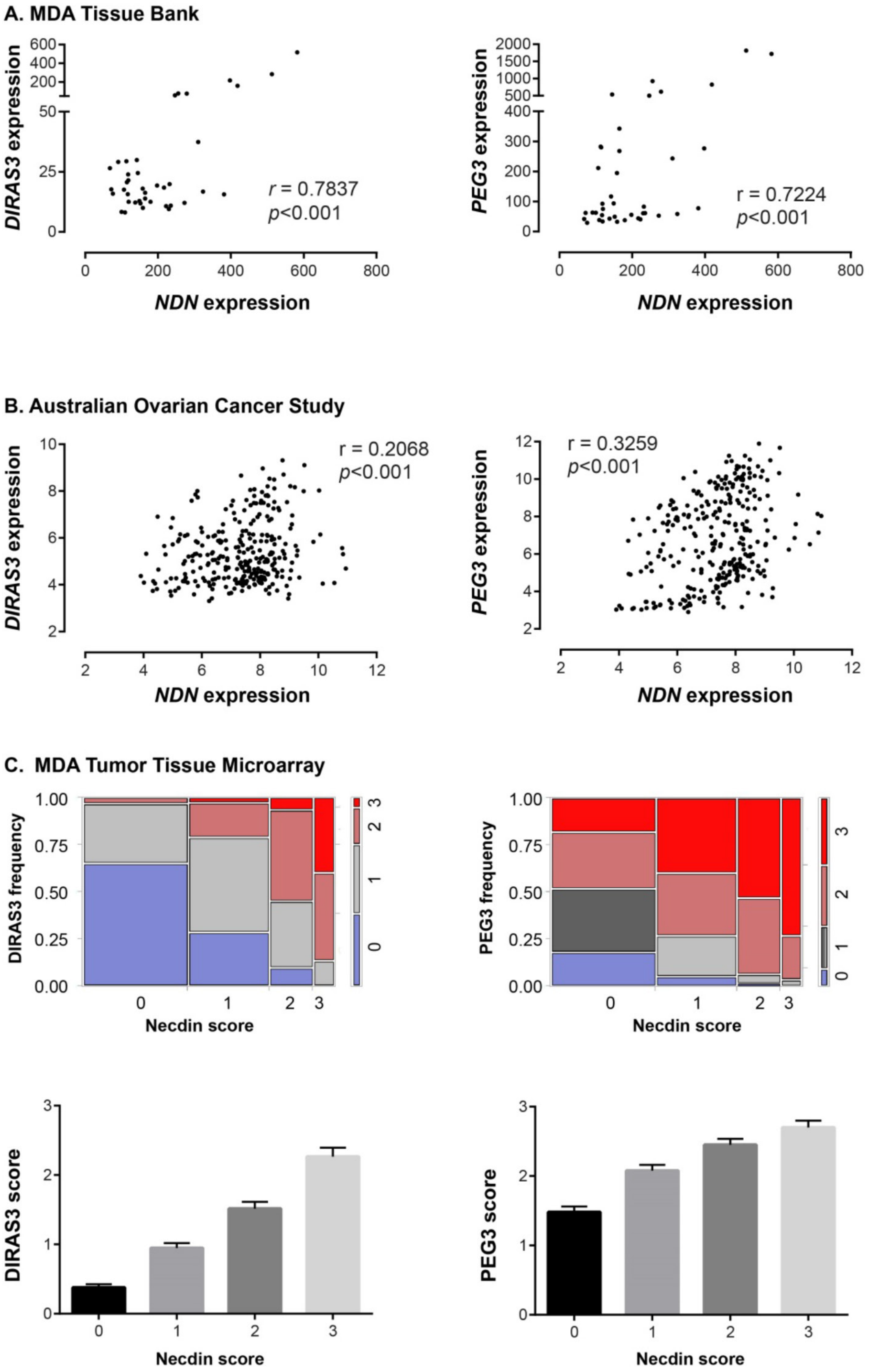

Supplementary Figure S3: *NDN* expression correlates with the expression of *DIRAS3* and *PEG3*. A. Correlation of mRNA levels using 35 ovarian cancer samples from MD Anderson Cancer Center Gynecological Tissue Bank; **B.** Correlation of mRNA levels using 295 ovarian cancer samples from AOCS database; **C.** Correlation of protein levels using ovarian cancer tissue microarrays generated by the Pathology Core of MD Anderson SPORE in Ovarian Cancer. The correlation in A and B was analyzed using Pearson correlation. Mosaic plot in C was generated using JMP software.

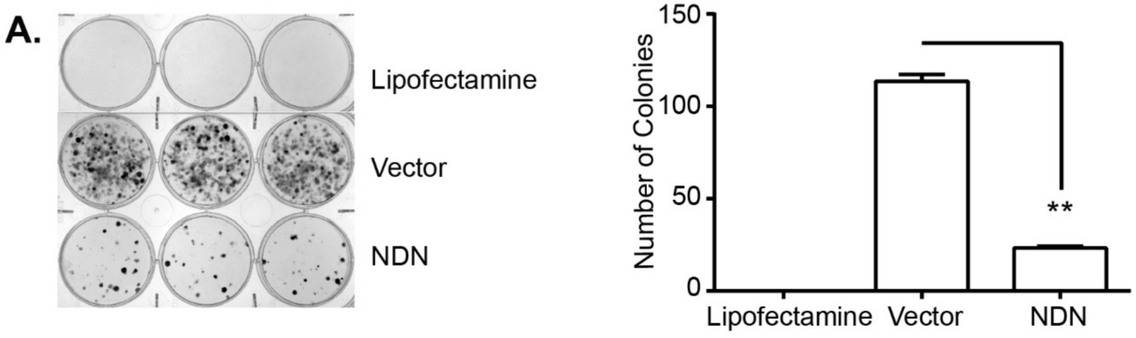

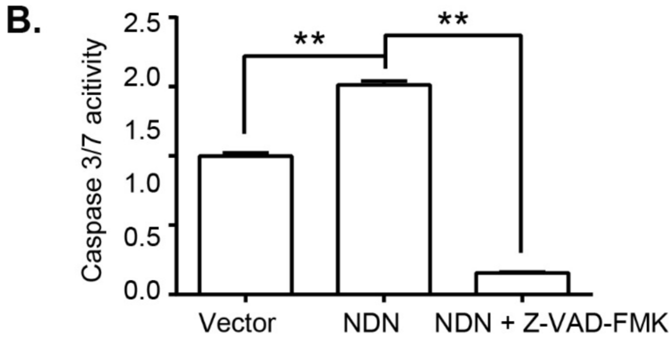

Supplementary Figure S4: *NDN* re-expression inhibits cell growth by inducing apoptosis in HEY cell line. A. *NDN* re-expression inhibits the colony formation. HEY A8 cells were transiently transfected with either empty vector or the plasmid containing *NDN* cDNA. After 24 hrs, cells were reseeded into 6-well plates at 1:16 dilution. G418 was added the next day to select transfected cells. Cells were allowed to grow for 2 weeks until the colonies were visible. Media were changed every other day. **B.** *NDN* re-expression increases caspase 3 and 7 activity. HEY cells were transfected with *NDN* for 48 hrs and Caspase 3/7 activity was measured using Caspase-Glo 3/7 assay kit from Promega.

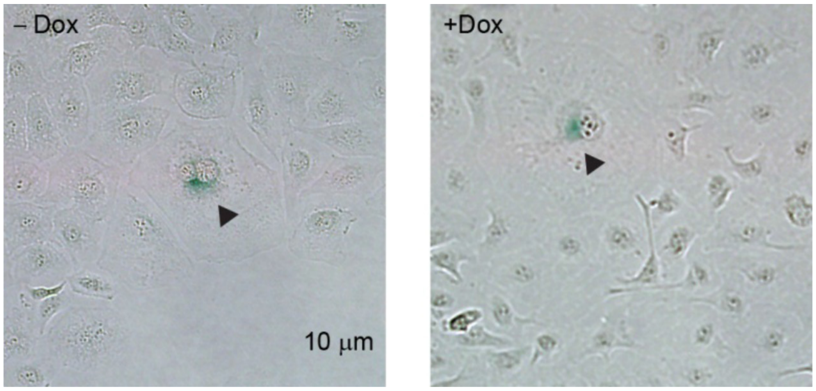

Supplementary Figure S5: *NDN* re-expression does not induce senescence in SKOv3ip-*NDN* inducible cells. Cells were incubated with or without Dox for 48 hrs and then stained with Senescence Cell Histochemical Staining Kit by Sigma.

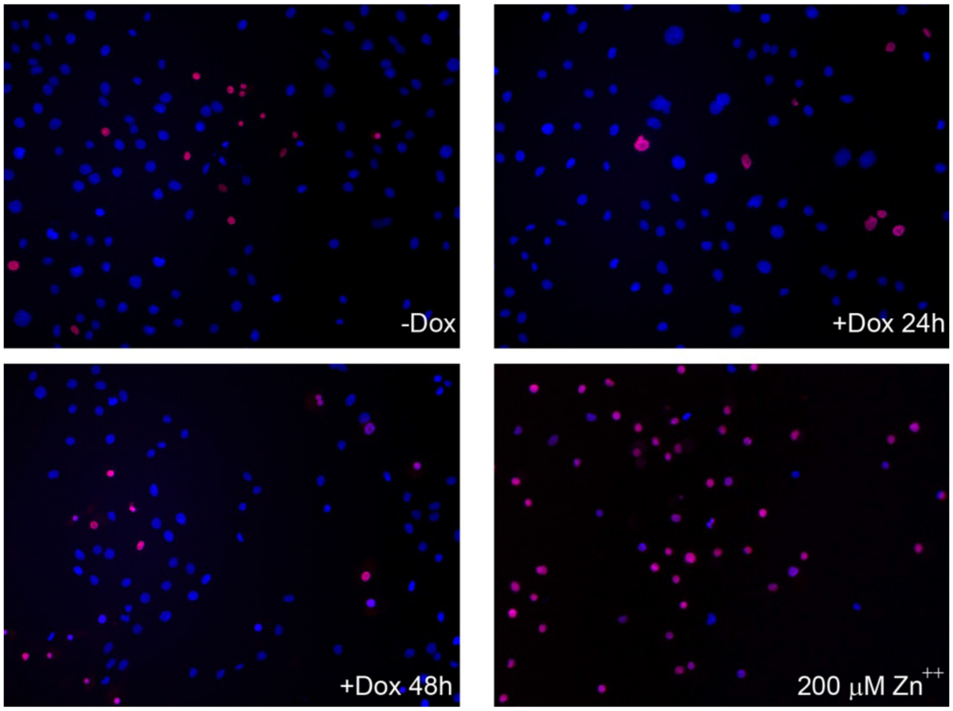

Supplementary Figure S6: *NDN* re-expression does not induce necrosis in SKOv3ip-*NDN* inducible cells. Cells were incubated with or without Dox and then stained with propidium iodide (PI) in red and Hoechst 33342 in blue. Zn^{2+} induced necrosis serves as a positive control.

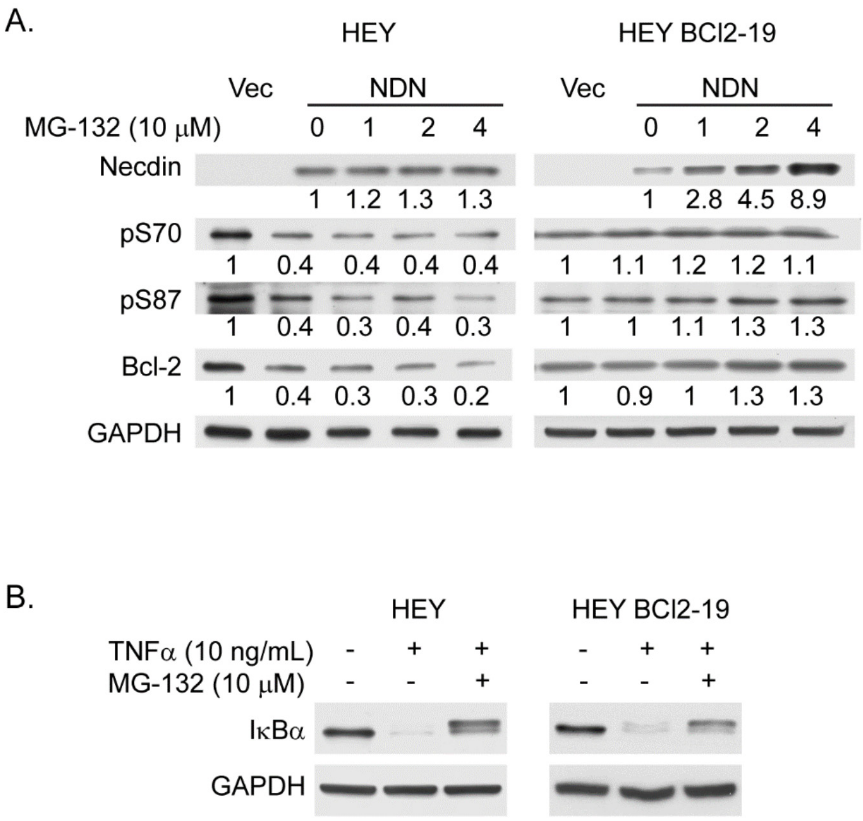

Supplementary Figure S7: Effects of *NDN* re-expression on Bcl-2. A. Proteasome inhibitor MG-132 does not restore Bcl-2 expression in WT HEY cells. HEY or HEY Bcl2-19 cell lines were transfected with empty vector or *NDN* plasmid for two days. Cells were then treated with 10 µM MG-132 and lysed at different time points. Cell lysates were resolved on 4-15% gradient SDS-PAGE gel and blotted with antibodies. **B.** MG-132 blocks proteasomal degradation of IκBα. Cells were treated with MG-132 for 2 hours prior to TNFα stimulation. Cells lysates were resolved on 10% SDS-PAGE gels and blotted for IκBα, which serves as a control for MG-132.

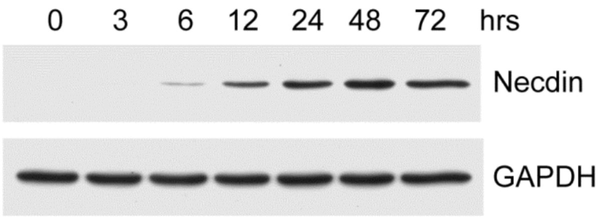

Supplementary Figure S8: Time course of *NDN* re-expression in *NDN*-7. *NDN* stable clone 7 was induced by doxycycline and harvested at different time points. Cell lysates were resolved on 10% SDS-PAGE gel and blotted with anti-necdin and anti-GAPDH antibodies.

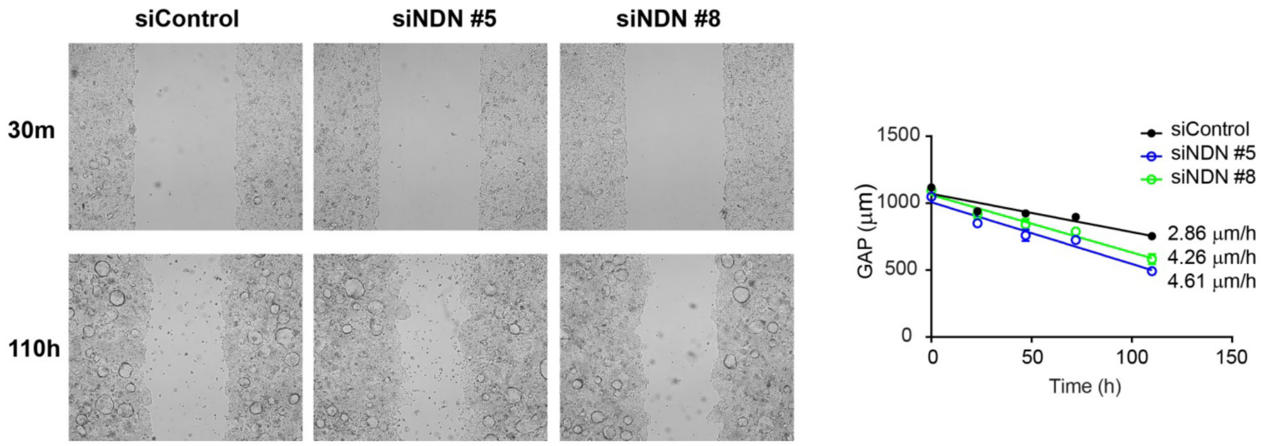

Supplementary Figure S9: NDN depletion slightly promotes cell motility. OVCA 432 cells were transfected with NDN siRNA. Following a scratch, the wound closure was recorded every 12 hrs up to 120 hrs. The migration speed was calculated by dividing gap width over time.

Supplementary Table S1: Culture media for 10 ovarian cancer cell lines and primary culture of normal ovarian epithelium

Cell line	Culture medium	Supplement
CaOv3	DMEM<br>(Mediatech 10017CV)	200mM L-Glutamine (MT25005CI) 6ml<br>100mM Sodium Pyruvate 6ml<br>10K PEN/STREP (MT30002CI) 6ml<br>Hyclone FBS 50ml
SKOv3	Macoy's 5A<br>(Mediatech 10050CV)	200mM L-Glutamine (MT25005CI) 6ml<br>10K PEN/STREP (MT30002CI) 6ml<br>Hyclone FBS 50ml
HEY<br>OVCAR3<br>OVCAR5<br>OC316	RPMI1640<br>(Mediatech 15040CV)	200mM L-Glutamine (MT25005CI) 6ml<br>100mM Sodium Pyruvate 6ml<br>10K PEN/STREP (MT30002CI) 6ml<br>Hyclone FBS 50ml (Thermo SV30014.03)
NOE	MCDB105 Medium<br>(Sigma M6295-10×1L);<br>Midium 199 (Mediatech 10060CV)<br>(1:1)	200mM L-Glutamine (MT25005CI) 6ml<br>10K PEN/STREP (MT30002CI) 6ml<br>Hyclone FBS 50ml<br>10ug/mL EGF (Sigma E4127-0.1MG) 0.5ml
DOV13<br>OVCA 420<br>OVCA 432<br>OVCA 433	MEM<br>(Mediatech 10-010-CV)	200mM L-Glutamine (MT25005CI) 6ml<br>10K PEN/STREP (MT30002CI) 6ml<br>100mM Sodium Pyruvate 6ml<br>Non-essential amino acids 6ml<br>Vitamin 6ml<br>Hyclone FBS 50ml
NDN inducible cell lines	Macoy's 5A<br>(Mediatech 10050CV)	200mM L-Glutamine (MT25005CI) 6ml<br>10K PEN/STREP (MT30002CI) 6ml<br>Hyclone FBS 50ml<br>G418 200μg/ml<br>Puromycin 0.5μg/ml
Ovarian epithelial primary culture	MCDB105 Medium<br>(Sigma M6295-10×1L)<br>Midium 199<br>(Mediatech 10060CV)<br>(1:1)	200mM L-Glutamine (MT25005CI) 6ml<br>10K PEN/STREP (MT30002CI) 6ml<br>Hyclone FBS 50ml<br>10ug/mL EGF (Sigma E4127-0.1MG) 0.5ml

Supplementary Table S2: Antibodies and reagents

Antibodies and reagents	Species	Vendor	Catalog
BAX antibody	Mouse	Santa Cruz	Sc-20067
Bcl-2 antibody	Mouse	Santa Cruz	Sc-509
Bcl-2 S70 antibody	Rabbit	Cell Signaling	2827
Bcl-2 S87 antibody	Rabbit	Santa Cruz	Sc-16323-R
Cleaved caspase 3 antibody	Rabbit	Millipore	AB3623
Cleaved caspase 7 antibody	Rabbit	Cell Signaling	8438
FAK pY397 antibody	Rabbit	Cell Signaling	8556
FAK pY925 antibody	Rabbit	Cell Signaling	3284
Total FAK antibody	Rabbit	Cell Signaling	3285
GAPDH antibody	Mouse	Millipore	MAB374
LC3B antibody	Rabbit	Cell Signaling	2775
Necdin antibody	Mouse	Novus	H00004692-M07
Necdin control IgG2a kappa	Mouse	Novus	NBP1-43318
Necdin Recombinant protein		Novus	H00004692-P01
Paxillin antibody	Rabbit	Cell Signaling	12065
RhoA antibody	Rabbit	Cell Signaling	2117
XIAP antibody	Mouse	BD Bioscience	610762
Oregon Green 488 phalloidin		Invitrogen	O7466

Supplementary Table S3: Pathologic features of ovarian cancer samples for Affymetrix array analysis

Histology	No.
Serous	17
Mucinous	5
Clear cell	4
Endometrial	9
Total	35

Supplementary Table S4: Patient characteristics of paired samples for LOH and DNA methylation

Clinical and pathologic features	No.
Stage	
I	7
II	4
III	22
IV	10
Histology	
Serous	18
Endometrial	5
Clear cell	3
Granulosa cell	3
Transitional cell	1
Mixed	13

Supplementary Table S5: Summary of methylation, LOH and expression in paired normal and tumor specimens

Supplementary Table S6: Methylation profile in paired normal and tumor specimens

	**Methylation level**	**CpG 1**	**CpG 2**
Promoter region	Average normal[a]	35.6 ± 4.8	41.6 ± 5.8
	Average tumor[b]	38.7 ± 14.3	44.1 ± 15.4
	% Hyper[1]	23%	30%
	% Normal[2]	65%	51%
	% Hypo[3]	12%	19%
Imprinting Center	Methylation level	CpG A	CpG B
	Average normal[a]	49.8 ± 4.4	42.7 ± 6.0
	Average tumor[b]	48.4 ± 14.9	39.3 ± 13.4
	% Hyper[1]	16%	5%
	% Normal[2]	63%	79%
	% Hypo[3]	21%	16%

[a]Average methylation levels (%) in normal tissues detected by pyrosequencing.
[b]Average methylation levels (%) in paired tumor tissues detected by pyrosequencing.
[1]Percentage of tumor specimens in which methylation levels are at least 2 standard deviation above normal specimens.
[2]Percentage of tumor specimens in which methylation levels are between 2 standard deviation from normal specimens.
[3]Percentage of tumor specimens in which methylation levels are at least 2 standard deviation below normal specimens.
