# Supplementary material for: NDN is an imprinted tumor suppressor gene that is downregulated in ovarian cancers through genetic and epigenetic mechanisms
Source: Oncotarget. 2015 Dec 12;7(3):3018–32. doi: 10.18632/oncotarget.6576 (PMC4823087; doi:10.18632/oncotarget.6576)
Supplement: Supplementary file 2 [file oncotarget-07-3018-s002.docx]

**Table S5.** Summary of methylation, LOH and expression in paired normal and tumor specimens

|  |  | **NDN Promoter** | | **Imprinting Center** | |  |  |
| --- | --- | --- | --- | --- | --- | --- | --- |
|  | **ID** | **CpG1** | **CpG2** | **CpGA** | **CpGB** | **LOH** | **Relative Expression to NOE** |
| 1 | 00-021 |  |  |  |  | homo | 0.1300 |
| 2 | 00-036 | **­** | **­** | **↑­** |  | **hetero** | 0.3000 |
| 3 | 00-039 | **­↑** | **↑** | **­** |  | **LOH** | 0.0710 |
| 4 | 00-055 |  | **­↑** |  |  | homo | 0.0500 |
| 5 | 00-066 |  |  |  |  | homo | 0.0000 |
| 6 | 00-107 |  | **­↑** |  |  | **hetero** | 0.0200 |
| 7 | 00-113 |  | **↓** |  | **­** | homo | 0.2700 |
| 8 | 00-126 | **↓** | **↓** | **↓** | **↓** | homo | 0.0300 |
| 9 | 00-136 |  |  |  | **↓** | homo | 0.1377 |
| 10 | 00-273 |  |  |  |  | homo | 0.0900 |
| 11 | 00-289 | **↓** | **↓** | **↓** | **↓** | **LOH** | 0.0100 |
| 12 | 01-002 | **­↑** | **­↑** | **­↑** |  | homo | 0.0300 |
| 13 | 01-003 |  | **↓** | **↑** | **­↑** | **hetero** | 0.0000 |
| 14 | 01-020 | **­↑** | **­↑** |  |  | **hetero** | 0.0200 |
| 15 | 01-118 |  |  | **↓** | **↓** | homo | 0.0800 |
| 16 | 01-161 |  | **↓** | **↓** |  | **hetero** | 0.0000 |
| 17 | 02-072 | **­↑** | **­↑** |  |  | homo | 0.0100 |
| 18 | 02-199 | **­↑** | **­↑** |  |  | homo | 0.0100 |
| 19 | 02-208 | **↓** | **↓** | **↓** |  | **LOH** | 0.0800 |
| 20 | 02-228 |  |  |  |  | homo | 2.1861 |
| 21 | 02-278 |  |  |  |  | homo | 0.7497 |
| 22 | 02-305 |  |  |  |  | homo | 0.0100 |
| 23 | 03-048 |  |  |  |  | homo | 0.0000 |
| 24 | 03-049 |  |  |  |  | **LOH** | 0.0000 |
| 25 | 03-055 |  |  | **­↑** |  | homo | 1.6671 |
| 26 | 03-069 |  |  |  |  | homo | 0.0900 |
| 27 | 03-102 |  |  |  |  | **hetero** | 8.3667 |
| 28 | 03-134 | **­↑** | **­↑** | **­↑** | **↓** | **hetero** | 0.0100 |
| 29 | 03-144 | **↓** | **↓** | **↓** |  | homo | 0.0300 |
| 30 | 03-147 | **­↑** | **­** | **­↑** |  | **hetero** | 0.0600 |
| 31 | 03-219 |  |  | **↓** |  | **LOH** | 0.1600 |
| 32 | 03-226 | **↓** | **↓** |  | **↓** | **hetero** | 2.7505 |
| 33 | 03-233 |  |  |  | **­** | **hetero** | 0.0100 |
| 34 | 03-296 |  |  |  |  | **hetero** | 0.3328 |
| 35 | 03-305 | **­↑** | **­↑** |  |  | **hetero** | 0.0100 |
| 36 | 03-309 | **­↑** | **­↑** | **­↑** | **­↑** | homo | 0.0686 |
| 37 | 04-059 |  |  |  |  | homo | 5.5318 |
| 38 | 04-094 |  |  |  |  | homo | 2.3058 |
| 39 | 04-113 | **­** | **↑­** |  |  | homo | 0.0000 |
| 40 | 04-139 |  |  | **↓** |  | homo | 0.0000 |
| 41 | 04-151 |  |  |  |  | **hetero** | 0.0100 |
| 42 | 04-162 | **­↑** | **­↑** |  | **­** | homo | 0.0200 |
| 43 | 04-172 | **­** | **­↑** | **↓** | **↓** | homo | 0.4300 |
| Count (%) | **­↑** | 10 (23%) | 13 (30%) | 7 (16%) | 2 (5%) |  |  |
|  | **↓** | 5 (12%) | 8 (19%) | 9 (21%) | 7 (16%) |  |  |
